# The interleukin-33 receptor contributes to pulmonary responses to ozone in male mice: role of the microbiome

**DOI:** 10.1186/s12931-019-1168-x

**Published:** 2019-08-27

**Authors:** David I. Kasahara, Jeremy E. Wilkinson, Youngji Cho, Aline P. Cardoso, Curtis Huttenhower, Stephanie A. Shore

**Affiliations:** 1 Molecular and Integrative Physiological Sciences Program, Department of Environmental Health, Harvard T.H. Chan School of Public Health, 665 Huntington Av Bld1 room 319, Boston, MA 02115 USA; 2 Department of Biostatistics, Harvard T.H. Chan School of Public Health, 665 Huntington Ave, Boston, MA 02115 USA

**Keywords:** IL-33, Sex differences, Airway responsiveness, Neutrophil, Interleukin-5

## Abstract

**Background:**

Interleukin-33 is released in the airways following acute ozone exposure and has the ability to cause airway hyperresponsiveness, a defining feature of asthma. Ozone causes greater airway hyperresponsiveness in male than female mice. Moreover, sex differences in the gut microbiome account for sex differences in this response to ozone. The purpose of this study was to determine whether there were sex differences in the role of interleukin-33 in ozone-induced airway hyperresponsiveness and to examine the role of the microbiome in these events.

**Methods:**

Wildtype mice and mice genetically deficient in ST2, the interleukin-33 receptor, were housed from weaning with either other mice of the same genotype and sex, or with mice of the same sex but opposite genotype. At 15 weeks of age, fecal pellets were harvested for 16S rRNA sequencing and the mice were then exposed to air or ozone. Airway responsiveness was measured and a bronchoalveolar lavage was performed 24 h after exposure.

**Results:**

In same-housed mice, ozone-induced airway hyperresponsiveness was greater in male than female wildtype mice. ST2 deficiency reduced ozone-induced airway hyperresponsiveness in male but not female mice and abolished sex differences in the response to ozone. However, sex differences in the role of interleukin-33 were unrelated to type 2 cytokine release: ozone-induced increases in bronchoalveolar lavage interleukin-5 were greater in females than males and ST2 deficiency virtually abolished interleukin-5 in both sexes. Since gut microbiota contribute to sex differences in ozone-induced airway hyperresponsiveness, we examined the role of the microbiome in these ST2-dependent sex differences. To do so, we cohoused wildtype and ST2 deficient mice, a situation that allows for transfer of microbiota among cage-mates. Cohousing altered the gut microbial community structure, as indicated by 16S rRNA gene sequencing of fecal DNA and reversed the effect of ST2 deficiency on pulmonary responses to ozone in male mice.

**Conclusions:**

The data indicate that the interleukin-33 /ST2 pathway contributes to ozone-induced airway hyperresponsiveness in male mice and suggest that the role of interleukin-33 is mediated at the level of the gut microbiome.

**Electronic supplementary material:**

The online version of this article (10.1186/s12931-019-1168-x) contains supplementary material, which is available to authorized users.

## Background

IL-33 is an alarmin stored within the nuclei of many cells including airway epithelial cells [1]. Upon cellular injury, IL-33 is released into the extracellular milieu where it activates cells bearing the IL-33 receptor, ST2. Such cells include type 2 innate lymphoid cells (ILC2s), γδ T-cells, mast cells, and T helper cells [2–7]. Genome-wide association studies show that both IL-33 and ST2 are associated with asthma [8–10]. Furthermore, in animal models, exogenous administration of IL-33 causes airway hyperresponsiveness (AHR) [11, 12], a canonical feature of asthma, and studies using IL-33 deficient or ST2 deficient mice indicate that IL-33 contributes to both allergic and virally-induced AHR [13, 14].

Ozone is a common air pollutant that can trigger asthma attacks [15–17]. Ozone inhalation causes lung and airway epithelial cell damage [18], leading to cytokine and chemokine release, neutrophil recruitment, and AHR [19–23]. Ozone also causes release of IL-33 into the airways [24]. We have reported that anti-ST2 antibodies do not attenuate ozone-induced AHR in female C57BL/6 mice [24]. However, the magnitude of ozone-induced AHR is greater in male than female mice [25, 26], and the role of IL-33 in male C57BL/6 mice has not been established. Importantly, ozone causes ST2-dependent activation of ILC2s and subsequent release of type 2 cytokines within the airways [24, 27, 28] and there are also sex differences in the number of ILC2s within the airways [29].

We have previously reported that the magnitude of ozone-induced AHR is regulated by gut microbiota: both antibiotic treatment and germ free conditions reduced ozone-induced AHR and inflammatory cell recruitment in male C57BL/6 mice [4]. Moreover, we observed sex differences in the gut microbial community structure [25], consistent with other reports [30]. We also reported that gut microbiota contribute to sex-differences in ozone-induced AHR: antibiotic treatment abolished sex differences in ozone-induced AHR [25]. Importantly, others have reported differences in the gut microbiomes of IL-33-deficient versus wildtype mice [31]. Taken together, the data suggest that sex differences in the role of IL-33 in ozone-induced AHR could be mediated through changes in the gut microbiome.

The purpose of this study was to examine sex differences in the role of IL-33 in ozone-induced AHR. To do so, we compared the effect of ST2 deficiency in male and female mice housed, from weaning, with other mice of the same genotype and sex. When the mice were 15 weeks old, they were exposed either to room air or to ozone. Our data indicated that ST2 deficiency reduced ozone-induced AHR and cellular inflammation in male but not in female mice, even though ST2 deficiency reduced the release of IL-33-dependent cytokines such as IL-5 in both sexes. To examine the role of the microbiome in these ST2-dependent sex differences, we cohoused WT and ST2 deficient mice. Cohousing altered the gut microbial community structure as indicated by 16S rRNA gene sequencing of fecal DNA and also reversed the effect of ST2 deficiency on pulmonary responses to ozone. The data indicate that in male mice, the role of IL-33 in responses to ozone is mediated not via its effects on cytokine release from ST2 bearing immune cells within the lungs, but rather via effects on the microbiome.

## Methods

### Mice

ST2 deficient mice (ST2^−/−^) on a C57BL/6 background were generated by Dr. Andrew McKenzie at Cambridge University [32] and obtained from a colony at Yale School of Medicine courtesy of Dr. Ruslan Medzhitov. ST2^−/−^ mice were crossbred with C57BL/6 J mice purchased from The Jackson Laboratories (Bar Harbor, ME) to generate heterozygous (ST2^+/−^) mice. ST2^+/−^ mice were crossed to generate most of the ST2^−/−^ and WT mice used for these experiments. Some WT and ST2^−/−^ offspring of the ST2^+/−^ parents were themselves used as breeders to obtain other WT and ST2^−/−^ mice used in these experiments. Breeders were fed mouse chow 5058 (PicoLab mouse diet 20, Lab Diet, St Louis, MO), but once weaned, the offspring used for experimentation were fed mouse chow 5053 (PicoLab mouse diet 20, Lab Diet). All experiments were approved by the Harvard Medical Area Animal Care and Use Committee.

Mice were genotyped at approximately 3 weeks of age. Mice were then segregated by sex and assigned to one of three caging schemes. “Same-housed” mice resided in cages hosting either WT mice with other WT mice or ST2^−/−^ mice with other ST2^−/−^ mice. Other WT and ST2^−/−^ mice were housed together (cohoused). Since mice ingest some of the fecal microbiota of their cagemates either during grooming or as a result of coprophagy, cohousing modifies the gut microbiota [31, 33]. Mice were same housed or cohoused for approximately 12 weeks after weaning. Cage changes for all mice were performed weekly by the same investigator.

### Protocol

Fecal pellets were collected immediately before exposure to air or ozone and fecal DNA extracted (see below). Same housed male and female mice were exposed to room air or to ozone (2 ppm for 3 h). Cohoused mice were only exposed to ozone. Twenty four hours later, mice were anesthetized and instrumented for the measurement of pulmonary mechanics and airway responsiveness to inhaled aerosolized methacholine as described below. Following these measurements, mice were euthanized with an overdose of sodium pentobarbital. A bronchoalveolar lavage was then performed to determine inflammation and injury.

### Ozone exposure

Mice were exposed to ozone (2 ppm) or ambient air for 3 h. During exposure, mice were housed individually in a wire mesh cage without food or water and placed inside a stainless steel and plexiglass exposure chamber. Ozone was generated by passing medical grade oxygen through an ozone generator and diluting with ambient air. Ozone concentration was constantly monitored during the exposure with an atmospheric gas analyzer (Model 49i, Thermo Scientific, Waltham, MA). At the end of exposure, food and water were restored and the mice were returned to clean cages.

### Measurement of pulmonary mechanics and airway hyperresponsiveness

Twenty-four hours after exposure, mice were anesthetized with an intra-peritoneal injection of sodium pentobarbital (50 mg/kg) and xylazine (7 mg/kg). Once the anesthetic plane was achieved, the trachea was isolated and cannulated with an 18G tube adapter, and connected to a computer-controlled ventilator (Flexivent, Scireq, Montreal, Canada). We performed a bilateral thoracotomy to expose the lungs to atmospheric pressure and we applied a positive expiratory-end pressure of 3 cmH_2_O. Measurements of baseline pulmonary resistance (R_L_) were followed by a dose-response to aerosolized methacholine (1–100 mg/mL in half log increments). R_L_ was measured using the forced oscillation technique, as previously described [34]. For each animal, we calculated the average of the 3 highest measurements of R_L_ at each dose and used these values to obtain dose-response curves.

### Bronchoalveolar lavage, ELISA, and multiplex analysis

The lungs were lavaged by instillation of 1 mL of ice cold PBS, twice. Lavage fluid was combined and centrifuged, the supernatant separated and stored at -80 °C, and the cells re-suspended in PBS. Total cells were counted with a hemocytometer. Cytospin slides were prepared and stained with Hemacolor (EMD-Millipore, Billerica, MA) to obtain differential counts. When available, at least 300 cells were counted. For multiplex assay, 500 uL of BAL supernatant was loaded into a 3kD filter (Amicon Ultra 3kda, EMD-Millipore, Bilerica, MA) and concentrated by centrifugation. The levels of pro-inflammatory cytokines and chemokines were determined in the concentrate via a multiplex assay (Eve Technologies, Calgary, Canada). The reported values have been corrected back to the original volume. BAL IL-17A (Biolegend, San Diego, CA) and IL-33 (eBiosciences, Waltham, MA)) were determined by commercial enzyme linked immunosorbent assay (ELISA) kits.

### Lung injury

Ozone-induced lung injury was determined by measurement of BAL protein, a marker of injury to the alveolar/capillary barrier [18]. The protein assay was performed using the Bicinchoninic acid method (Pierce-Thermo Fischer, Rockford, IL).

### Fecal DNA extraction

Fecal pellets were collected immediately before mice were exposed to air or ozone and total DNA was extracted from fecal pellets as follows. 80uL of sterile PBS was added to 15–30 mg of feces and homogenized by using a TissueLyzer (Qiagen, Valencia, CA). Lysis buffer and proteinase-K were added, and samples were incubated for at least 24 h at 57 °C with periodic vortexing. The samples were centrifuged to remove debris, and supernatant was transferred to a new tube and processed for total DNA isolation by using commercial columns (QIAmp DNA mini kit, Qiagen, Valencia, CA). Fecal DNA concentration and quality were measured with a Nanodrop (ThermoFisher, Waltham, MA).

### 16S rRNA sequencing and analysis

16S rRNA gene sequencing was performed on fecal DNA harvested from male mice. Sequencing was performed at the Massachusetts Host-Microbiome Center at Brigham and Women’s Hospital. Briefly, 5–10 ng of DNA was amplified by PCR at variable region 4 of the 16S rRNA gene with barcoded bacterial universal primers (515F and 806R) that contain Illumina MiSeq sequencing adaptors. Amplicon products were checked by electrophoresis and pooled to produce a library used for sequencing by MiSeq (Illumina, San Diego, CA). The resulting sequencing data (FastaQ) data were analyzed at Harvard T. H. Chan School of Public Health Microbiome Analysis Core as previously described [4] . Briefly, forward and reverse sequences were analyzed via UPARSE OTU algorithm for assembly of sequences, to remove chimera sequences, and to prepare taxonomic classification. The OTUs were classified against GreenGenes 16S RNA database version 13.8 for taxonomic prediction, and to generate OTU tables. The resulting OTU tables were then used to predict functional metagenomes via PICRUSt (Phylogenetic Investigation of Communities by Reconstruction of Unobserved States) [35]. Sequencing raw data (Fastaq) and metadata have been deposited at National Institute of Health – Sequence Read Archive (SRA) with accession number PRJNA516522 (sequences SAMN10790706–41).

### Microbial analysis via PCR

To compare the relative abundance of *Lactobacillus* (genus) and *Akkermansia muciniphila* in fecal DNA from female versus male mice, we used qPCR analysis quantified by SYBR green and normalized the data to total bacterial taxa via pan-bacterial primers and following analysis via the ΔΔCT method. Primer sequences used for this PCR were: Pan-bacterial (926F: 5′-AAACTCAAKGAATTGACGG-3′ K = G or T, 1062R: 5′-CTCACRRCGAGCTGA-3′, R = A or G [36]), *Lactobacillus* genus (Forward: 5′-AGCAGTAGGGAATCTTCCA-3′, Reverse: 5′-ATTYCACCGCTACACATG-3′, Y=C or T [37]), and *Akkermansia muciniphila* (Forward: 5′-CAGCACGTGAAGGTGGGGAC-3′, Reverse: 5′-CCTTGCGGTTGGCTTCAGAT-3′ [38]).

### Statistical analysis

Outlier analysis and exclusion were performed by using GraphPad Prism and Grubbs test. Statistical analysis of lung mechanics, protein assay, ELISA and multiplex cytokines were performed by using Factorial ANOVA with Fisher’s LSD as post-hoc test. BAL cells were log transformed before running the Factorial ANOVA in order to conform to a normal distribution. A *p* value < 0.05 was considered statistically significant. For the 16S sequencing data, we used Multivariate Association with Linear Model - MaAsLin [39] to assess significant associations at the per-feature level among both housing and genotype factors in arcsine-square root transformed relative abundance data, and only data with *p* < 0.05 and q < 0.25 were considered statistically significant. Statistical analysis of functional metagenomes predicted from 16S taxonomic data obtained through PICRUSt [35] was also performed with MaAsLin.

## Results

**Fig. 1:**
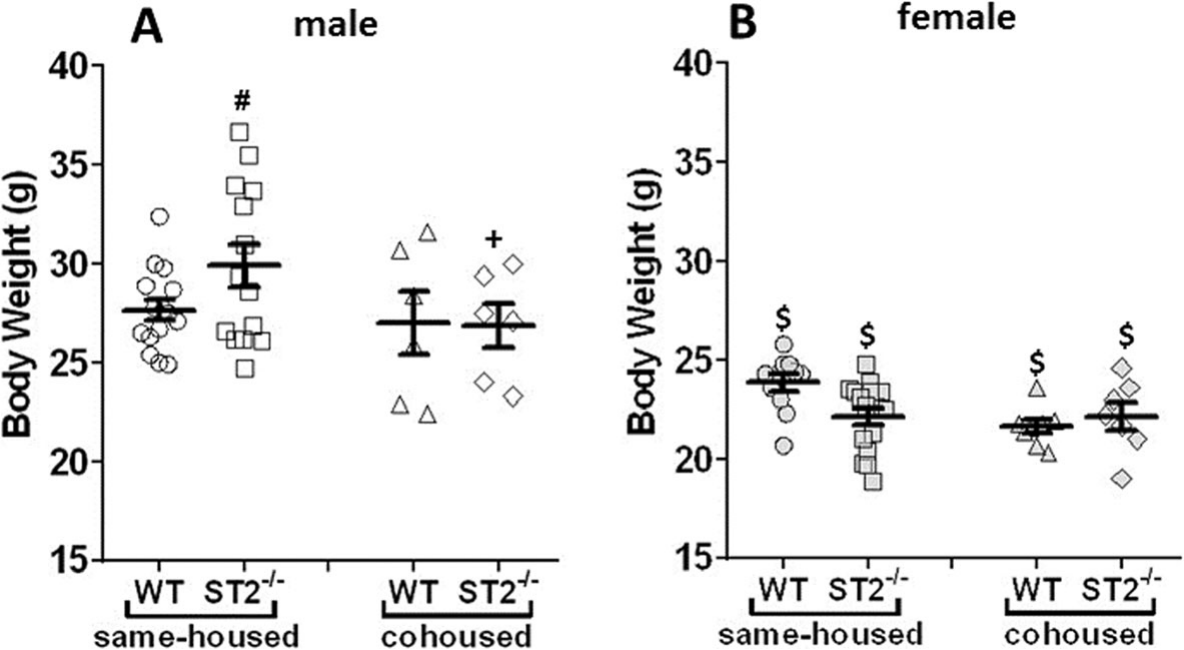
Body weights of male (**a**) and female (**b**) mice immediately before measurement of lung function. Mean ± SEM. # *p* < 0.05 vs WT, + *p* < 0.05 vs same-housed mice, and $ *p* < 0.05 vs male mice

Same-housed ST2^−/−^ male mice weighed slightly but significantly more than WT controls, and this difference was abolished in cohoused mice (Fig. 1). Female mice weighed less than males, but there was no effect of ST2 deficiency or cohousing in female mice.

Below, we first describe the effects of ST2 deficiency on pulmonary responses to ozone in same-housed mice. We then compare same-housed and cohoused mice.

### Effect of ST2 deficiency on pulmonary responses to ozone in same-housed mice

#### Effect of ST2 deficiency on ozone-induced AHR

**Fig. 2:**
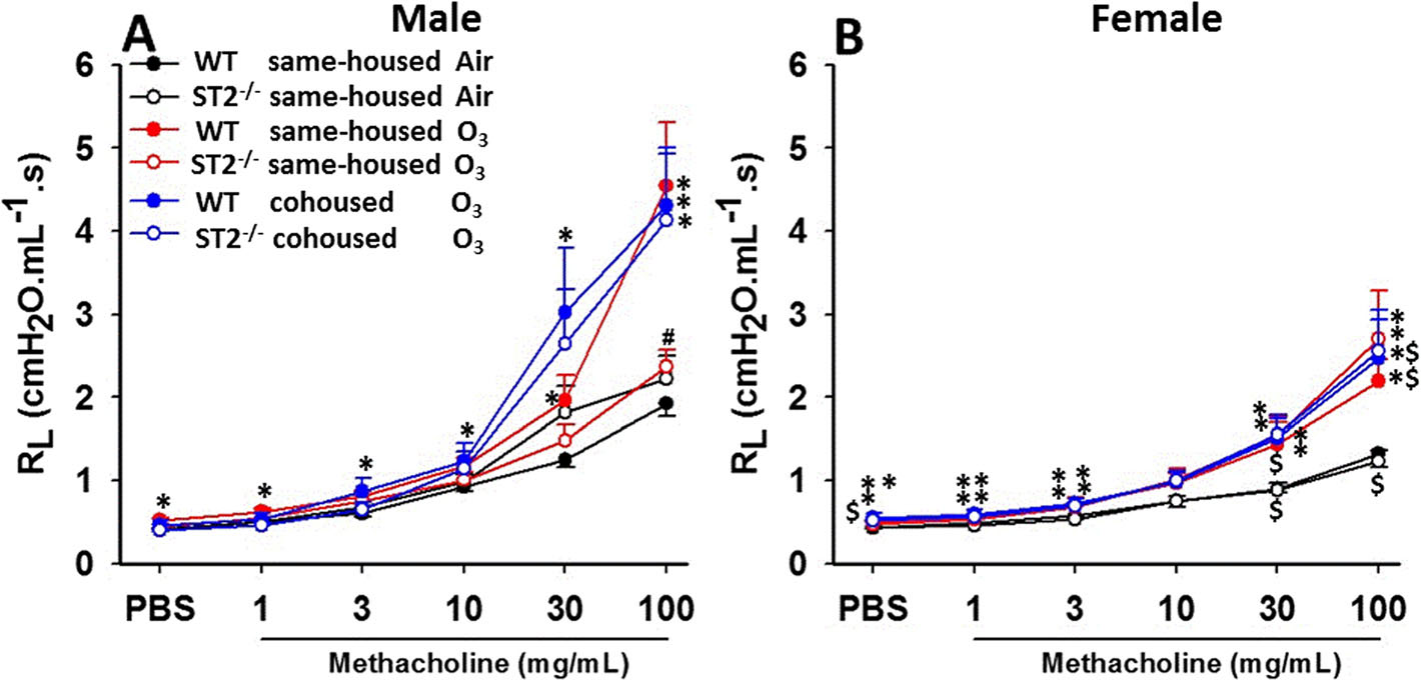
Effect of ST2 deficiency on ozone-induced airway hyperresponsiveness. Changes in pulmonary resistance (R_L_) induced by inhaled aerosolized methacholine were assessed in male (**a**) and female (**b**) wildtype (WT) and ST2 deficient (ST2^−/−^) mice exposed to air or ozone. Mice were either housed with other mice of the same genotype (same-housed) or with mice of the other genotype (cohoused). Data are expressed as mean ± SEM, *p < 0.05 vs Air, #*p* < 0.05 vs WT mice with the same exposure, $ *p* < 0.05 vs male mice. *N* = 6–11 per group for male mice and *N* = 5–8 per group for female mice

In same-housed mice exposed to air, ST2 deficiency had no effect on airway responsiveness regardless of sex (Fig. 2a, b). Compared to air-exposed WT male mice, ozone-exposed WT male mice developed AHR. Compared to WT male mice, ozone-induced AHR was attenuated in ST2^−/−^ mice (Fig. 2a). Compared to male WT mice, the magnitude of ozone-induced AHR was significantly lower in female WT mice, as previously reported by ourselves and others [25, 26]. In female mice exposed to ozone, there was no effect of ST2 deficiency on airway responsiveness (Fig. 2b). The net effect of these changes was such that in ST2^−/−^ mice, sex differences in the magnitude of ozone-induced AHR were no longer apparent.

#### Effect of ST2 deficiency on ozone-induced cellular inflammation and injury

**Fig. 3:**
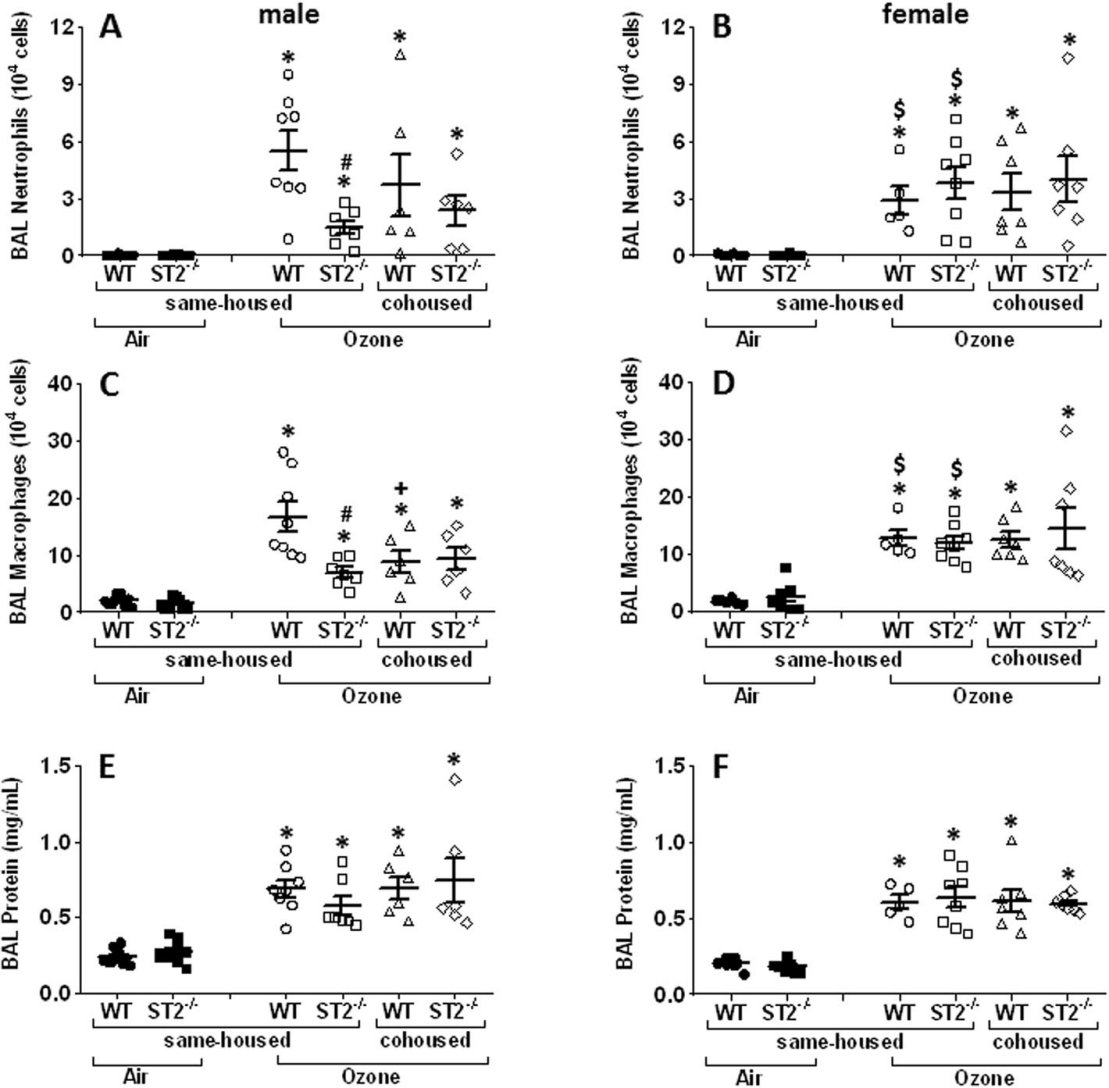
Effect of ST2 deficiency on ozone-induced inflammation and injury. Bronchoalveolar lavage (BAL) neutrophils (**a**, **b**), macrophages (**c**, **d**), and protein (**e**, **f**) in air and ozone-exposed male (**a**, **c**, **e**) and female (**b**, **d**, **f**) mice. Each symbol denotes one individual mouse. Horizontal and vertical lines indicate mean and SEM respectively. *p < 0.05 vs Air, **#** *p* < 0.05 vs WT, **+** *p* < 0.05 vs same-housed, **$** *p* < 0.05 vs male

In same- housed mice, ozone exposure increased BAL neutrophils and macrophages whether the mice were male or female and whether the mice were WT or ST2 deficient (Fig. 3a, b, c and d). Compared to WT male mice exposed to ozone, BAL neutrophils and BAL macrophages were attenuated in ST2^−/−^ male mice exposed to ozone (Fig. 3a ,c). This effect of ST2 deficiency was not related to changes in ozone-induced lung injury, since BAL protein, a marker of ozone-induced injury to the alveolar capillary barrier [40] was not affected by ST2 deficiency (Fig. 3e, f). In contrast to the effects of ST2 deficiency observed in male mice exposed to ozone, in female mice there was no effect of ST2 deficiency on BAL neutrophils or BAL macrophages (Fig. 3b, d). As in the male mice, BAL protein was unaffected by ST2 deficiency in female mice (Fig. 3f).

#### Effect of ST2 deficiency on ozone-induced increases in BAL cytokines and chemokines

**Fig. 4:**
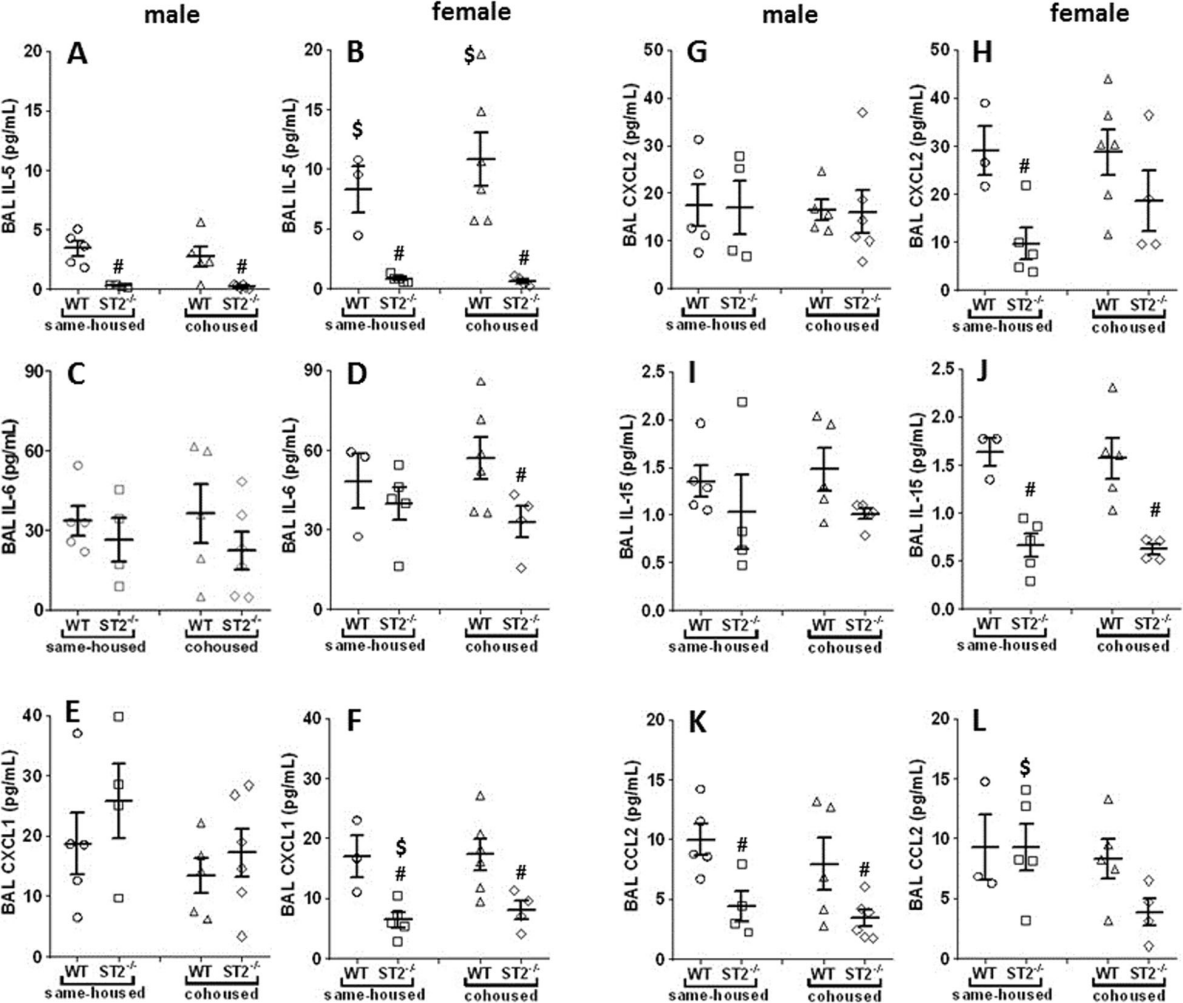
*Effect of ST2 deficiency on BAL cytokines and chemokines in ozone exposed mice.* Shown are BAL concentrations of IL-5 (**a**, **b**), IL-6 (**c**, **d**), CXCL1 (**e**, **f**), CXCL2 (**g**, **h**), IL-15 (**i**, **j**), and CCL2 (**k**, **l**) in male (**a**, **c**, **e**, **g**, **i**, **k**) and female (**b**, **d**, **f**, **h**, **j**, **l**) mice exposed to ozone. Each mouse is represented by an individual symbol. Horizontal and vertical lines indicate mean and SEM respectively. **#** *p* < 0.05 vs WT mice; **$** *p* < 0.05 vs male mice

To determine whether sex differences in the impact of ST2 deficiency on pulmonary responses to ozone (Figs. 1 and 2) were the result of sex differences in IL-33 release, we measured BAL concentrations of IL-33. In same-housed mice exposed to ozone, BAL IL-33 was not influenced by sex or by ST2 deficiency (Additional file 1: Figure S1). The effects on airway responsiveness of exogenously administered IL-33 or IL-33 release caused allergen or viruses is mediated, at least in part, by release of type 2 cytokines [13, 14]. We and others have established that ozone increases BAL concentrations of the type 2 cytokine, IL-5 [24, 41]. Hence, to determine whether there were sex differences in the *response* to IL-33 that might explain sex-differences in the effects of ST2 deficiency (Figs. 2 and 3), we measured BAL concentrations of IL-5. In same-housed mice, ST2 deficiency caused a marked and significant decrease in BAL IL-5 (Fig. 4a, b) in both male and female mice, consistent with the known effects of IL-33 in provoking type 2 cytokine release from ILC2s and other cells [7, 11, 42]. In WT mice, BAL concentrations of IL-5 were significantly greater in females than in males, even though the effect of ST2 deficiency on ozone-induced AHR and inflammatory cell recruitment was observed only in male mice (Figs. 2 and 3). The data indicate that sex differences in the activation of Th2 cytokine-producing cells by IL-33 do not account for the observed sexual dimorphism in pulmonary responses to ozone.

Exogenously administered IL-33 also causes the production of other cytokines and chemokines, including IL-6, CXCL1, and CXCL2 [43, 44]. Each of these cytokines is induced by ozone and is known to contribute to pulmonary responses to ozone [45–47]. Indeed, with the exception of IL-6, each of these cytokines was reduced by ST2 deficiency in female mice exposed to ozone (Fig. 4d, f, and j). However, there was no effect of ST2 deficiency on BAL concentrations of these cytokines in male mice and BAL concentrations of IL-5 were actually significantly higher in female than in male WT mice, even though only the male mice had reductions in ozone-induced AHR and inflammatory cell recruitment after ST2 knockout (Figs. 2 and 3). BAL concentrations of other cytokines and chemokines induced by ozone exposure, including IL-15, IL-17A, eotaxin, G-CSF, IL-9, IP-10, LIF, MIG, MIP-1α, and MIP-1β were not different in male and female WT mice (Fig. 4 i and j, and Additional file 1: Table S1). Of these, IL-15 was reduced by ST2 deficiency in female but not male mice (Fig. 4j) and CCL2 (MCP-1) was reduced by ST2 deficiency in male but not female mice (Fig. 4k and l). ST2 deficiency did not affect BAL concentrations of other cytokines and chemokines (Additional file 1: Table S1). Taken together, the data do not support the hypothesis that sex differences in cytokine release by IL-33 account for observed sex differences in the effect of ST2 deficiency on ozone-induced AHR and inflammatory cell recruitment.

### Effect of ST2 deficiency on pulmonary responses to ozone in cohoused mice

We have recently reported that the gut microbiome contributes to sex differences in ozone-induced AHR [4, 25]. Others have reported that there are differences in the gut microbiomes of IL-33^−/−^ versus WT mice and that cohousing WT and IL-33^−/−^ mice reduces differences in their gut microbiomes and ablates IL-33-dependent differences in susceptibility to colitis in these mice [31]. Taken together, these data suggest that the microbiome might contribute to the sex differences observed in ST2-dependent responses to ozone (Figs. 2 and 3). To address this hypothesis, we compared responses to ozone in the same-housed mice described above with those obtained in WT and ST2^−/−^ mice that had been cohoused. Since neither ST2 deficiency (Fig. 2) nor microbial depletion or microbial transfer [4, 25] had any effect on airway responsiveness in air-exposed same-housed mice, cohoused mice were only examined after ozone exposure.

Whereas ST2 deficiency reduced ozone-induced AHR in same housed male mice, there was no significant effect of ST2 deficiency on ozone-induced AHR in cohoused male mice (Fig. 2a). After ozone exposure, WT male mice housed with ST2^−/−^ male mice had the same airway responsiveness as WT mice cohoused with WT mice. However, compared to ST2^−/−^ same-housed mice which had lower ozone-induced AHR than WT same-housed mice, ST2^−/−^ cohoused mice had greater airway responsiveness (Fig. 2a). The data suggest that effects of ST2 deficiency on ozone-induced AHR are dependent on gut microbiota or their metabolites. No effect of cohousing was observed in either WT or ST2^−/−^ female mice.

Whereas ST2 deficiency reduced ozone-induced neutrophil and macrophage influx into the lungs in same-housed male mice, there was no significant effect of ST2 deficiency on BAL neutrophils or macrophages in cohoused male mice (Figs. 3a and c). As was the case for ozone-induced AHR, the data suggest that these effects of ST2 deficiency on ozone-induced cellular inflammation are dependent on gut microbiota or their metabolites. No effect of cohousing on inflammatory cells was observed in either WT or ST2^−/−^ female mice (Figs. 3b, d).

In contrast to the differences in the effect of ST2 deficiency on ozone-induced AHR and inflammatory cell recruitment described above for cohoused versus same-housed mice, we did not observe any differences in the effect of ST2 deficiency on BAL cytokines in cohoused versus same-housed mice. Those cytokines that were different in ST2^−/−^ versus WT same housed mice were also different in ST2^−/−^ versus WT cohoused mice (Figs. 4a, b, f, j and k). These data further support the hypothesis that effects of ST2 deficiency on ozone-induced AHR and inflammatory cell recruitment observed in same-housed male mice are related to effects on the microbiome rather than effects on ozone-induced cytokine release within the lungs.

### Effect of ST2 deficiency and cohousing on the community structure of the gut microbiome

**Fig. 5:**
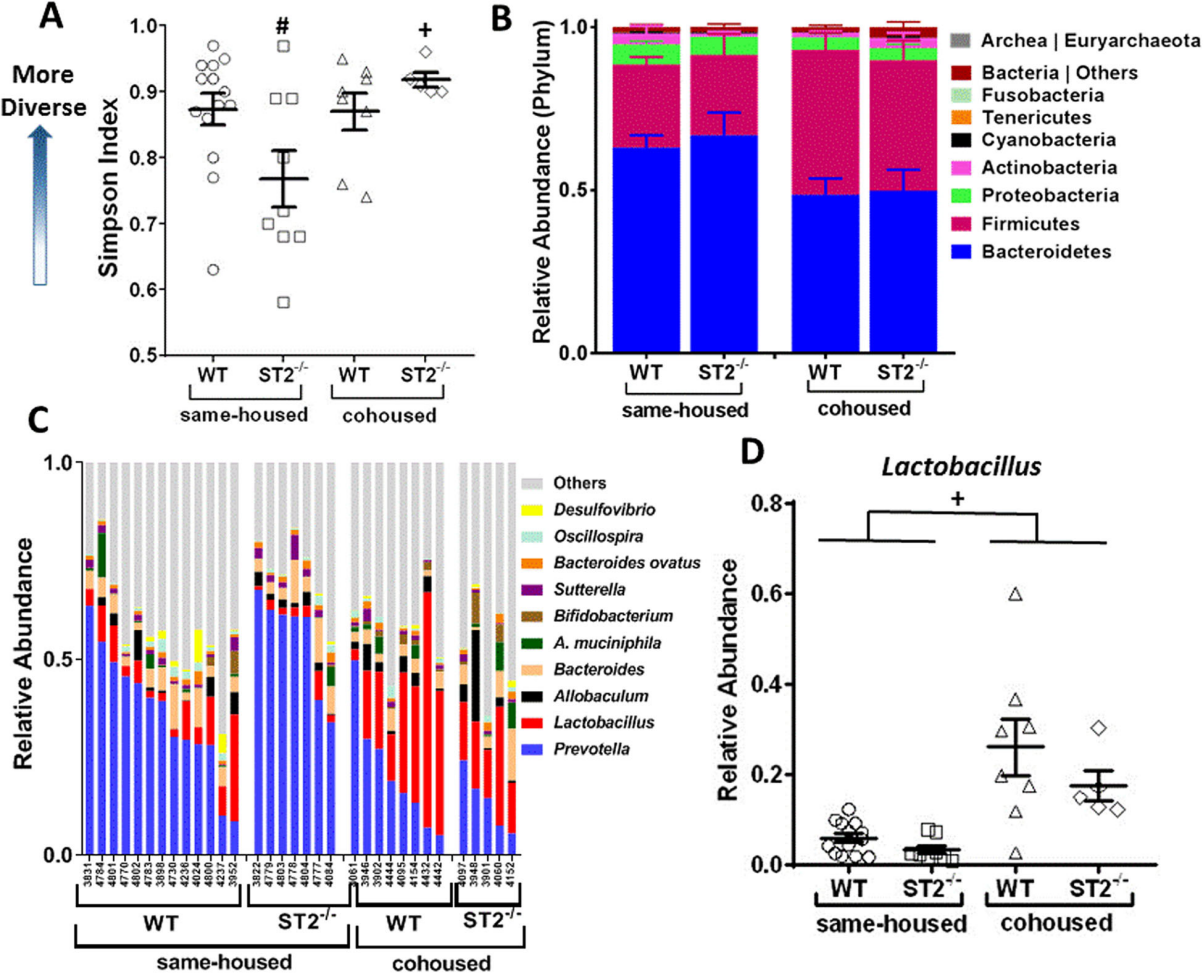
Effect of ST2 deficiency and cohousing on the gut microbiome of male mice. DNA was extracted from fecal pellets of male mice prior to exposure. Results of 16S rRNA sequencing of fecal DNA harvested from male WT and ST2^−/−^ same housed and cohoused mice. **a**) Simpson index; **b**) Taxa relative abundances at the phylum level; **c**) Top 10 genera taxa abundances relative to total gut Bacteria and Archaea reads. **d**) *Lactobacillus* genus determined by MaAsLin as significantly affected by housing (*p* = 0.0015, q = 0.196). In **a**, **b**, and **d** bars indicate mean ± SEM. In **a** and **d**, each symbol indicates one mouse. In **c**, each column indicates one mouse. **#** *p* < 0.05 vs WT mice, **+** *p* < 0.05 vs same-housed mice by Factorial ANOVA. Statistics were performed on arcsine-square root transformed data

Since cohousing reversed the effect of ST2 deficiency on ozone induced AHR only in male mice, we used 16S rRNA sequencing to determine the taxonomic composition of gut microbiota by analyzing fecal DNA obtained prior to exposure from same-housed and cohoused WT and ST2^−/−^ male mice. Since the fecal samples were collected before any exposure was administered, we combined sequencing data from fecal samples of mice that were ultimately exposed to air or to ozone. We did not perform 16S rRNA sequencing in female mice because we did not observe any effect of cohousing in these mice (Figs. 2 and 3). Analysis of 16S rRNA sequencing data was performed at the Harvard T.H. Chan School of Public Health Microbiome Analysis Core. We examined the gut rather than the lung microbiome because previous data from our lab suggest that the former rather than the latter accounts for the role of the microbiome in pulmonary responses to ozone [4]. The Simpson index, a measure of ecological diversity, indicated significantly reduced diversity (lower Simpson index) in ST2-deficient mice versus WT same housed mice (Fig. 5a). Cohousing reversed the impact of ST2 deficiency on microbial diversity.

**Fig. 6:**
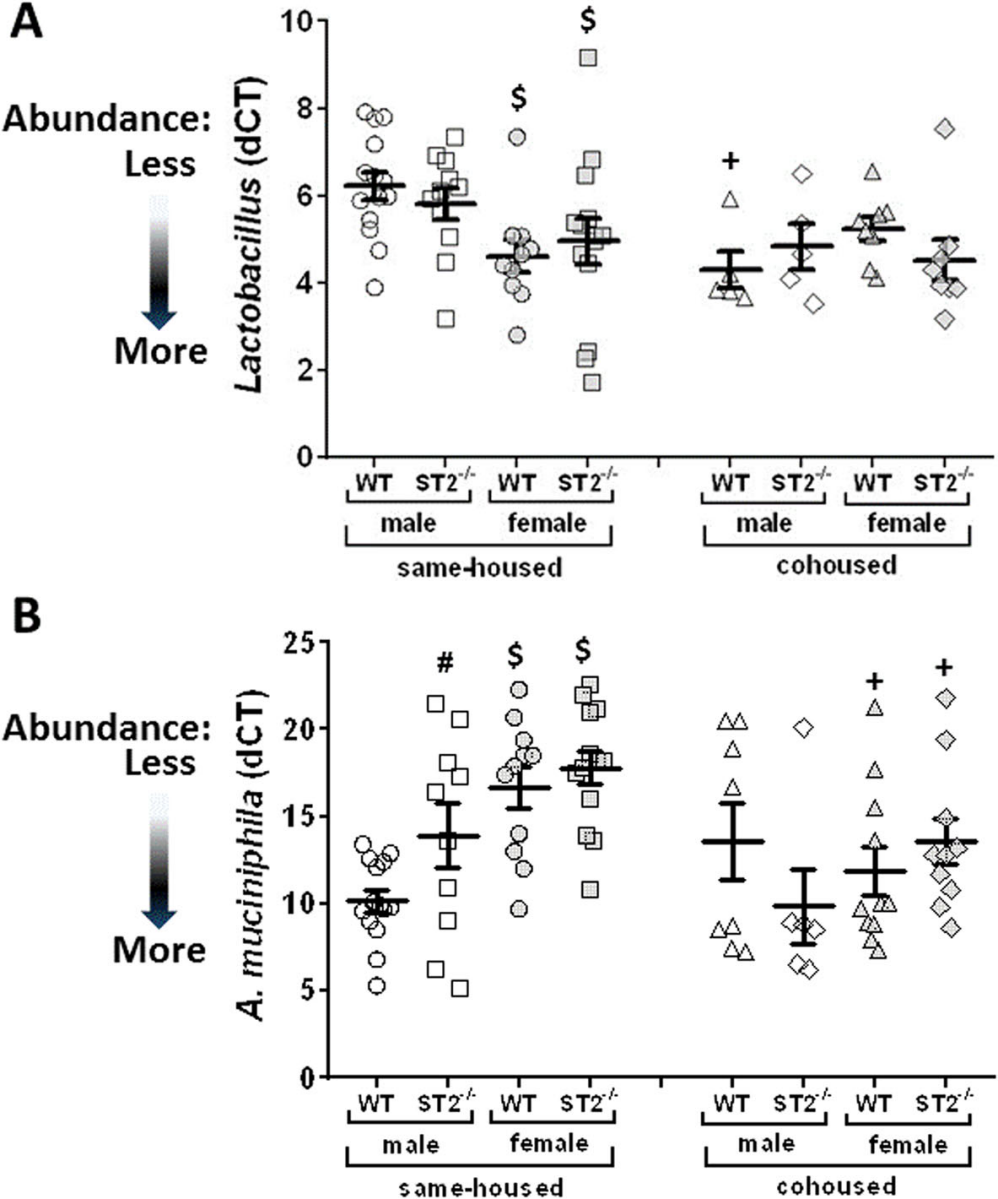
Effect of ST2 deficiency and cohousing on the abundances of Lactobacillus and Akkermansia muciniphila. Abundances of *Lactobacillus* (**a**) and *Akkermansia muciniphila* (**b**) were measured by qPCR of fecal DNA and normalized using pan bacterial primers. DNA was prepared from fecal pellets obtained from male and female, WT and ST2^−/−^, same-housed and cohoused mice immediately before the mice were exposed to air or ozone. Bars are mean ± SEM. Each symbol indicates one mouse. **#** *p* < 0.05 vs WT mice, **+** *p* < 0.05 vs same-housed mice, and **$** *p* < 0.05 vs male mice

Phylum level analysis indicated an increase in the abundance of Firmicutes in cohoused versus same housed mice whether the mice were WT or ST2 deficient (*p* < 0.05 in both cases, Fig. 5b). There was also a trend towards a decreased abundance of Bacteroidetes in cohoused versus same housed mice, although the effect did not reach statistical significance (Fig. 5b). More in depth analysis of the 10 most abundant taxa indicated additional effects of cohousing on the gut microbial community structure (Fig. 5c). In particular, there was a significant increase in the abundance of bacteria of the *Lactobacillus* genus in cohoused versus same housed mice (Figs. 5c, d, and 6a). This effect of cohousing was observed in both WT and ST2^−/−^ male mice and was confirmed by PCR-based analysis of the fecal DNA (Fig. 6a). In contrast, in female mice, the abundance of *Lactobacillus* was not affected by cohousing, likely because compared to male mice, the abundance of *Lactobacillus* was already significantly elevated even in the same-housed female mice (Fig. 6a). The overall increase in *Lactobacillus* genus (Firmicutes phylum) in cohoused versus same housed mice coincided inversely with an overall decrease in *Prevotella* (Bacteroidetes phylum) (Fig. 5c).

Others have reported differences in the abundance of *Akkermansia muciniphila (A. muciniphila)* in the gastrointestinal tracts of IL-33^−/−^ versus WT same-housed mice [31]. Moreover, these differences in *(A. muciniphila)* abundance appear to account for effects of IL-33 deficiency on susceptibility to colitis. Mice lacking IL-33 had worse DSS-induced colitis than WT mice, but this effect was reversed by cohousing the IL-33^−/−^ mice with WT mice [31]. Consequently, we considered the possibility that differences in *(A. muciniphila)* abundance might also account for differences in the susceptibility to ozone observed in mice deficient in the IL-33 receptor, ST2. The depth of our 16S rRNA sequencing analysis was not sufficient to determine whether there were genotype, cohousing, or sex-dependent differences in this very low abundance taxon, which was virtually undetectable by 16S rRNA sequencing in many samples. However, qPCR assay of *A. muciniphila* in fecal samples did indicate a significant decrease in the abundance of this taxon in male same-housed ST2^−/−^ versus WT mice, which reverted to WT levels after cohousing (Fig. 5b). Compared to same housed male WT mice, same-housed female WT mice also had lower abundance of *A. muciniphila,* but the abundance of this taxon was not affected by ST2 deficiency in female mice (Fig. 6b). However, cohousing increased the abundance of *A. muciniphila* in both WT and ST2^−/−^ female mice.

**Fig. 7:**
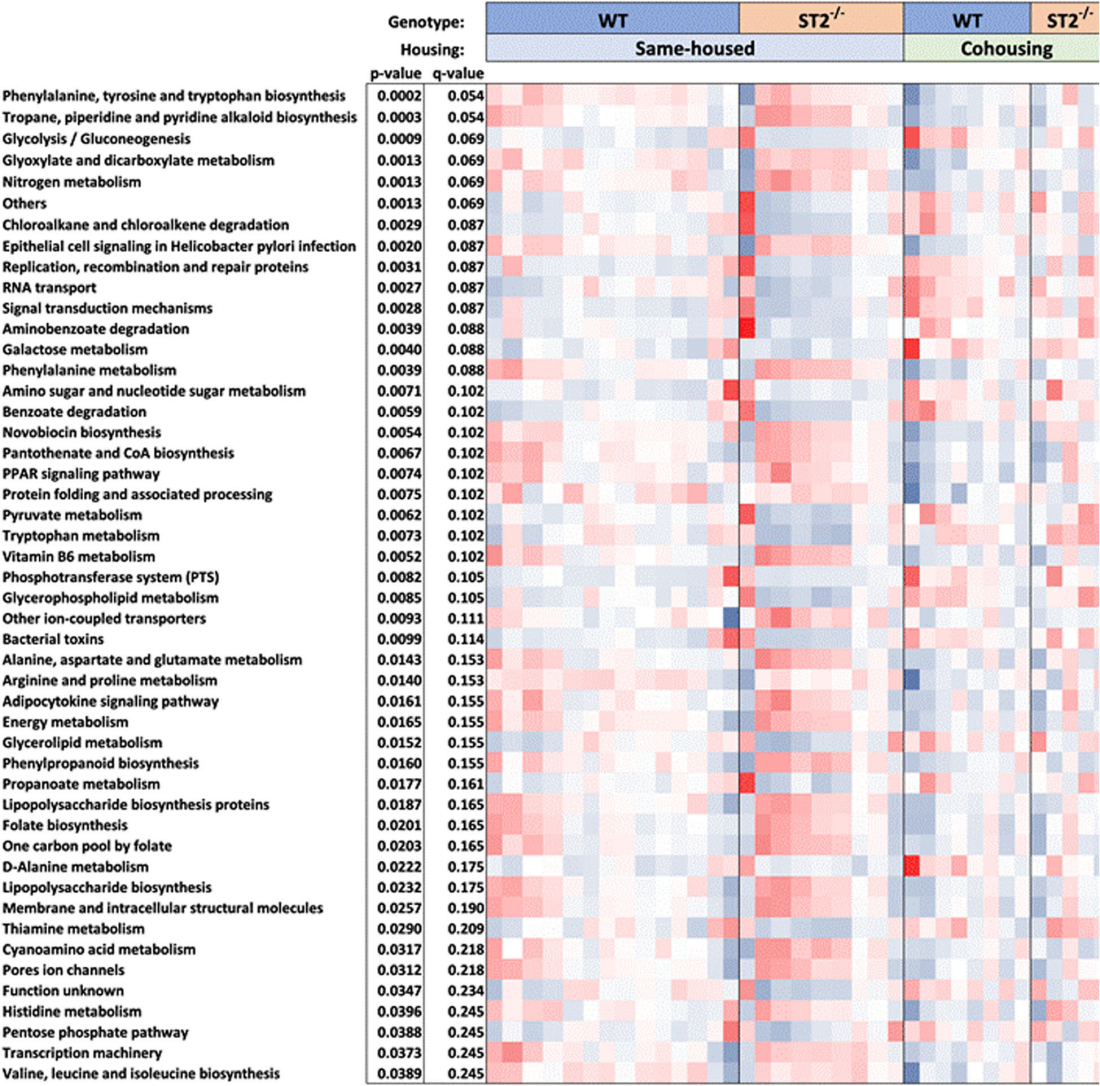
Predicted microbial functions affected by housing status in male mice. Heat map showing data derived from the gut taxonomic structure determined by 16S rRNA sequencing of DNA derived from fecal pellets harvested prior to exposure and were calculated by association of each taxon with KEGG predicted functions using PICRUSt [35]. Only data with *p* < 0.05 and q < 0.25 from arcsine transformed data comparing same-housed vs cohoused mice are included

**Fig. 8:**
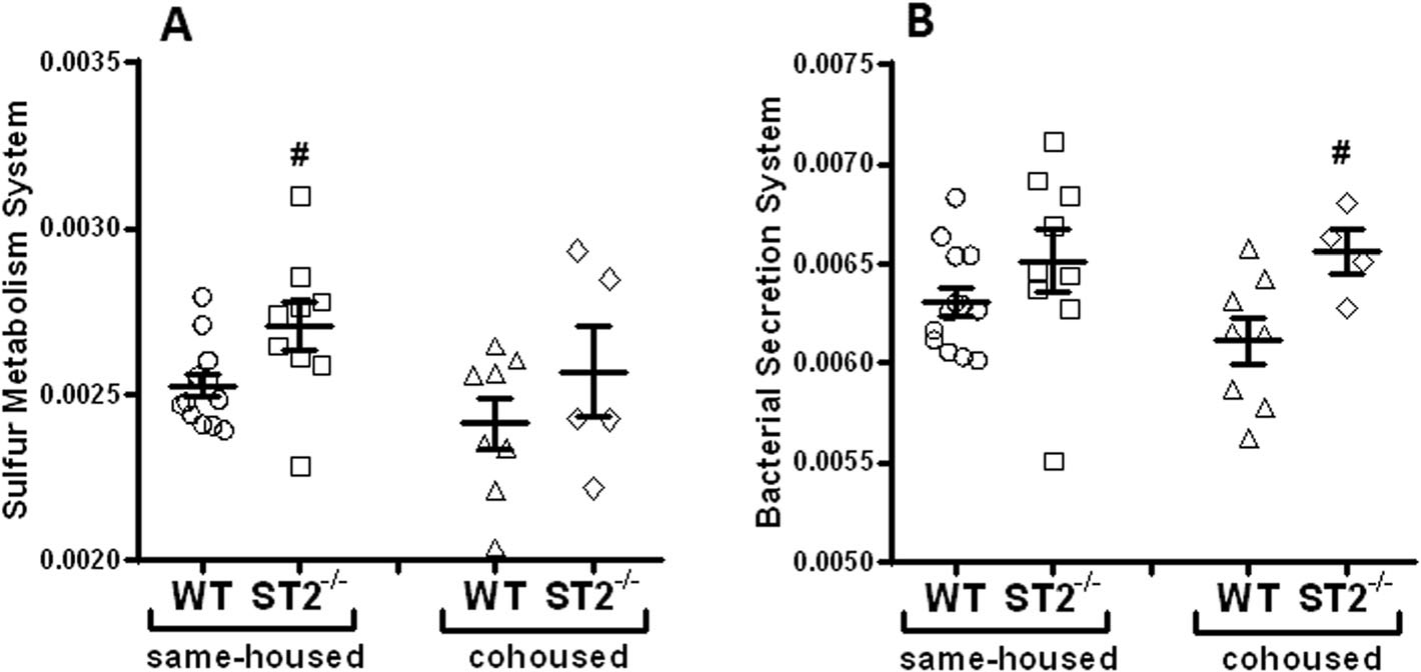
Predicted microbial functions affected by genotype in male mice. Data were generated as described in Fig. 8. Figures represent relative abundance of taxa involved in **a**) Sulfur Metabolism System (MaAsLin *p* = 0.020, Q = 0.165) and **b**) Bacterial Secretion System (MaAsLin *p* = 0.023, Q = 0.175). Grubbs test excluded outliers and Factorial ANOVA was performed on arcsine-square root transformed data. Bars represent mean ± SEM. Each symbol indicates 1 mouse. **#** *p* < 0.05 vs WT mice

The functional capacity of the gut microbiome may be as important for the health of the host as the particular identities of the taxa making up the gut microbial community structure [48–50]. PICRUSt (Phylogenetic Investigation of Communities by Reconstruction of Unobserved States) is a method that correlates each OTU (Operational Taxonomic Unit) determined by 16S rRNA gene sequencing to predicted KEGG (Kyoto Encyclopedia of Genes and Genomes) curated functions [35]. Our data indicated that in male mice, regardless of genotype, cohousing caused significant changes (*p* < 0.05 and q < 0.25) in 48 of 152 function/metabolite pathways analyzed (Fig. 7). Given the cohousing-induced alteration in the abundance of *Lactobacillus* (genus) observed in male mice (Figs. 5c, d and 6a), it is not surprising that many of the KEGG functions significantly affected by cohousing, were those in which lactate is one of substrates (Glycolysis/Gluconeogenesis, Pyruvate metabolism, Glyoxylate metabolism, Valine/Leucine/Isoleucine biosynthesis, and Propanoate metabolism (Fig. 7)). In contrast to the substantial effects of cohousing, MaAsLin analysis of PICRUSt data indicated that only two pathways were significantly affected by genotype: sulfur metabolism and bacterial secretion system (Fig. 8). ST2 deficient mice had a significantly higher index of sulfur metabolism than WT mice (Fig. 8a), and this effect was mainly driven by the same-housed mice, a pattern similar to that observed for ozone-induced AHR. Bacterial secretion system was also affected by ST2 deficiency, but only in cohoused mice (Fig. 8b).

## Discussion

Our data indicated that ozone-induced AHR and cellular inflammation were reduced in male but not female mice that were genetically deficient in the IL-33 receptor, ST2. Remarkably, these reduced responses to ozone were only observed when the mice were housed with other mice of the same genotype and not when WT and ST2^−/−^ mice were cohoused, a situation that allows for transfer of microbiota among cage-mates. Indeed, significant differences in the gut microbiome, particularly the abundance of *Lactobacillus* were observed in same-housed versus cohoused male mice. The data indicate that the IL-33/ST2 pathway contributes to ozone-induced AHR and cellular inflammation in male mice, and suggest that the role of IL-33 is mediated at the level of the gut microbiome.

Our data indicated sex differences in the magnitude of ozone-induced AHR (Fig. 2), consistent with previous reports [25, 26]. Notably, under same-housed conditions, ST2 deficiency attenuated O_3_-induced AHR in male but not female mice and virtually abolished these sex differences (Fig. 2). Nevertheless, ST2 deficiency caused a marked reduction in BAL IL-5 in both males and females confirming the activation of the IL-33 pathway and the efficacy of the ST2 knockdown in both sexes. Indeed, BAL IL-5 was greater in ozone-exposed WT female than WT male mice, consistent with reports of others that the abundance of ILC2s, one of the cell types responsible for type 2 cytokine production after ozone exposure, is greater in lungs of female than male mice [29, 51]. The data indicate a dissociation between type 2 cytokines and ozone-induced AHR. BAL CXCL1 and CXCL2 were also reduced by ST2 deficiency in female but not male mice. Taken together, the data suggest that activation of type 2 cytokine producing cells does not account for ST2-dependent effects on ozone-induced AHR observed in male same-housed mice. The observation that reductions in ozone-induced AHR and cellular inflammation in ST2^−/−^ versus WT male mice were not observed when the mice were cohoused, even though BAL IL-5 was still reduced, also supports this hypothesis.

Instead of effects mediated at the level of lung immune cell activation, our data support the hypothesis that the role of the IL-33/ST2 pathway was mediated via effects on the gut microbiome. Cohousing the WT and ST2^−/−^ male mice altered the gut microbiome (Figs. 5, 6 and 7) and abolished the impact of ST2 deficiency on ozone-induced AHR and inflammation observed in male mice (Figs. 2 and 3). Whether the ST2-dependent effects on the microbiome also account for the role of IL-33 in the AHR induced by allergen and by virus [13, 14] remains to be determined.

A role for the microbiome in ST2-mediated effects on responses to ozone is also consistent with the observed sex differences in the impact of ST2 deficiency. We observed sex differences in the gut microbiome (Fig. 6), consistent with other reports [25, 30, 52] as well as sex differences in the effect of ST2 deficiency on the microbiome (Fig. 6b). Furthermore, in female mice, cohousing did not impact bacterial taxa that were affected by cohousing in male mice (Fig. 6a), nor did cohousing impact ozone-induced AHR in female mice (Fig. 2b). A role for the microbiome in these events is also consistent with previous reports from our lab indicating that ozone-induced AHR and cellular inflammation are reduced by antibiotics or germ-free conditions in male mice [4] and that antibiotics abolish sex differences in ozone-induced AHR [25]. Moreover, a male-like pattern of increased responsiveness to ozone is induced in female mice by housing them in cages conditioned by male mice [25]. Because mice are coprophagic, such cage conditioning transfers the feces of the male to the females. As discussed above, our data suggest that *Lactobacillus* may be among the bacteria taxa that confer these sex differences. 16S rRNA sequencing of the fecal DNA from female mice might identify additional taxa.

One limitation of the study is that although 16S rRNA sequencing identified significant differences in *Lactobacillus* in cohoused versus same housed male mice (Fig. 5c, d), 16S rRNA sequencing did not identify significant differences in any taxa in ST2^−/−^ versus WT male mice. Nevertheless, there was a decrease in bacterial diversity (reduction in Simpson index) in same-housed ST2^−/−^ versus WT mice that was abolished in cohoused ST2^−/−^ versus WT mice (Fig. 5a), consistent with the reduction in ozone-induced AHR that was observed in same-housed ST2^−/−^ versus WT mice but abolished in cohoused ST2^−/−^ versus WT mice (Fig. 2). PCR of fecal DNA also indicated reductions in *A. muciniphila* in same-housed ST2^−/−^ versus WT mice that were abolished in cohoused ST2^−/−^ versus WT mice (Fig. 6b). It is conceivable that either or both of these changes contributed to the role of the microbiome in ST2-dependent responses to ozone observed in male mice. Nevertheless, others have proposed that it is not the taxonomic composition of the gut microbiome but rather changes in the functional capacity of the microbiome that impact the health of the host: many different taxa have the same functional capacities [49, 53]. We noted marked differences in the functional capacity of the microbiome in cohoused versus same housed mice (Fig. 7) that included changes in propanoate metabolism. We have previously reported a role for bacterial metabolism of short chain fatty acids, including propionate, in ozone-induced AHR in male mice [4]. Hence, it is conceivable that changes in this functional category could account for differences in ozone-induced AHR observed in same housed and cohoused mice (Fig. 2a). However, only two KEGG functional categories were affected by ST2 deficiency: sulfur metabolism and bacterial secretion system (Fig. 8). Of these, only the pattern of change in sulfur metabolism corresponded to the pattern of change in ozone-induced AHR: a significant increase in same housed ST2^−/−^ versus same housed WT mice that was abolished in cohoused mice. The change in sulfur metabolism is particularly interesting given that hydrogen sulfite, one end product of sulfur metabolism, abrogates ozone-induced AHR and attenuates ozone-induced neutrophilic inflammation in mice [54]. Thus, an increase in the capacity for sulfur metabolism in the gut microbiome of same housed ST2^−/−^ mice might be expected to result in reduced AHR and neutrophilic inflammation, as observed (Figs. 2 and 3).

Our data indicated a significantly greater abundance of bacteria of the *Lactobacillus* genus in same-housed female versus male mice (Fig. 6a), consistent with other reports by ourselves and others [25, 55, 56]. The magnitude of ozone-induced AHR was also reduced in female versus male WT mice (Fig. 2), consistent with our previous report [25]. Coupled with data showing that administration of probiotics containing bacteria of the *Lactobacillus* genus attenuates allergen-induced AHR [57] and also attenuates the ability of particulate air pollution to exacerbate allergen-induced AHR [58], the data suggest a role for lactobacilli or their metabolites in suppressing airway responsiveness. Data from human subjects point toward a link between lactobacilli and allergic asthma [59, 60], though the mechanistic basis for this relationship remains to be established. Whereas differences in the abundance of lactobacilli may account for sex differences in the magnitude of ozone-induced AHR, such differences do not appear to explain the ability of cohousing to ablate ST2-dependent reductions in ozone-induced AHR observed in male mice: lactobacilli were more abundant in both WT and ST2^−/−^ cohoused versus same housed mice (Fig. 6a), but cohousing only impacted ozone-induced AHR in ST2^−/−^ mice (Fig. 2).

A strength of this study was the breeding strategy. Since we predominantly bred ST2^+/−^ mice, most of the WT and ST2^−/−^ mice were derived from the same litters. Thus, the environmental conditions extant in the cages of the WT and ST2^−/−^ mice from birth to weaning were the same and the mice were inoculated at birth with the same microbiome. Differences in the gut microbiomes of these mice identified when the mice were approximately 15 weeks of age (Figs. 5a, 6a, b and 8) are therefore the result of effects related to ST2 deficiency rather than to changes that resulted as breeding colonies of WT and ST2^−/−^ mice stochastically diverged. In this context, it is interesting to note that, whereas we observed no effect of ST2 deficiency on ozone-induced changes in BAL neutrophils, BAL protein, or airway responsiveness in female mice, Michaudel et al. reported that ST2 deficiency augments ozone-induced changes in these outcome indicators in female mice, suggesting a protective role for IL-33 in the setting of ozone exposure [61]. The community structure of the gut microbiome is strongly impacted by housing conditions and is known to vary across animal housing facilities [52, 62, 63]. Given the role of the microbiome in pulmonary responses to ozone [4, 25] and data reported here indicating that both ST2 deficiency and cohousing can impact the gut microbiome (Figs. 5, 6, 7 and 8), the most likely explanation for the difference in the impact of ST2 deficiency in our study versus that of Michaudel et al. [61] is differences in the gut microbiome. Studies in germ free ST2 deficient mice or in ST2 deficient mice treated with antibiotics would permit evaluation of non-microbiome dependent effects of IL-33 that impact responses to ozone. Our data also emphasize the need for attention to mouse housing conditions and the microbiome in any study of the impact of genetic deficiencies.

## Conclusions

In conclusion, our data indicate that the IL-33/ST2 pathway is involved in ozone-induced AHR and cellular inflammation in male but not female mice. Importantly, our data also demonstrate that at least in these lean male mice, the role of this pathway is mediated not via its effect on cytokine release from ILC2s and other immune cells within the lungs, but rather via its effect on the microbiome. These observations may have important implications for the development of pharmaceuticals that target the IL-33/ST2 pathway and suggest the need for sex-specific therapeutics.

## Additional file

Additional file 1: **Table S1.** BAL cytokine and chemokine concentrations measured 24 h after ozone exposure. **Figure S1.** BAL IL-33 in male and female mice. (DOCX 48 kb)

## Data Availability

Sequencing raw data (Fastaq) and metadata have been deposited at National Institute of Health – Sequence Read Archive (SRA) with accession number PRJNA516522 (sequences SAMN10790706–41). Other datasets used and analyzed in this study are available from the corresponding author on reasonable request.

## Abbreviations

AHR: Airway hyperresponsiveness
ANOVA: Analysis of variance
BAL: Bronchoalveolar lavage
CCL2: Chemokine (C-C motif) ligand 2
CXCL: Chemokine (C-X-C motif) ligand
ELISA: Enzyme linked immunosorbent assay
G-CSF: Granulocyte-colony stimulating factor
IL: Interleukin
ILC: Innate lymphoid cell
IP-10: Interferon gamma-induced protein 10 (Cxcl9)
KEGG: Kyoto Encyclopedia of Genes and Genomes
LIF: Leukemia inhibitor factor
MaAsLin: Multivariate Association with Linear Model
MCP-1: Monocyte chemoattractant protein 1
MIG: Monokine Induced by Gamma interferon
MIP: Macrophage inflammatory protein
OTU: Operational Taxonomic Unit
PCR: Polymerase chain reaction
PICRUSt: Phylogenetic Investigation of Communities by Reconstruction of Unobserved States
qPCR: Quantitative polymerase chain reaction
R_L_: Pulmonary resistance
rRNA: Ribosomal RNA
ST2: Interleukin 1 receptor-like 1
WT: Wildtype
