# Supplementary material for: The interleukin-33 receptor contributes to pulmonary responses to ozone in male mice: role of the microbiome
Source: Respir Res. 2019 Aug 27;20:197. doi: 10.1186/s12931-019-1168-x (PMC6712741; doi:10.1186/s12931-019-1168-x)
Supplement: Supplementary file 1 — Table S1. BAL cytokine and chemokine concentrations measured 24 h after ozone exposure. Figure S1. BAL IL-33 in male and female mice. (DOCX 48 kb) [file 12931_2019_1168_MOESM1_ESM.docx]

Additional file 1

ST2-dependent pulmonary responses to ozone in male mice: role of the microbiome

David I. Kasahara^1^, Jeremy E. Wilkinson^2^, Youngji Cho^1^, Aline P. Cardoso^1^, Curtis Huttenhower^2^, Stephanie A. Shore^1^

1. Molecular and Integrative Physiological Sciences Program, Department of Environmental Health; 2. Department of Biostatistics, Harvard T.H. Chan School of Public Health, 665 Huntington Ave., Boston, MA, 02115

**Table of Contents:**

**Table S1:** BAL cytokine and chemokine concentrations measured 24 hours after ozone exposure.

**Figure S1**: BAL IL-33 in male and female mice.

**Table S1.** BAL cytokine and chemokine concentrations measured 24 hours after ozone exposure.

Results are mean ± SE of data from 6-7 mice per group for IL-17A and 3-6 mice per group for other cytokines and are expressed in pg/ml. IL-17A was determined by ELISA. Other cytokines and chemokines were determined by multiplex assay.


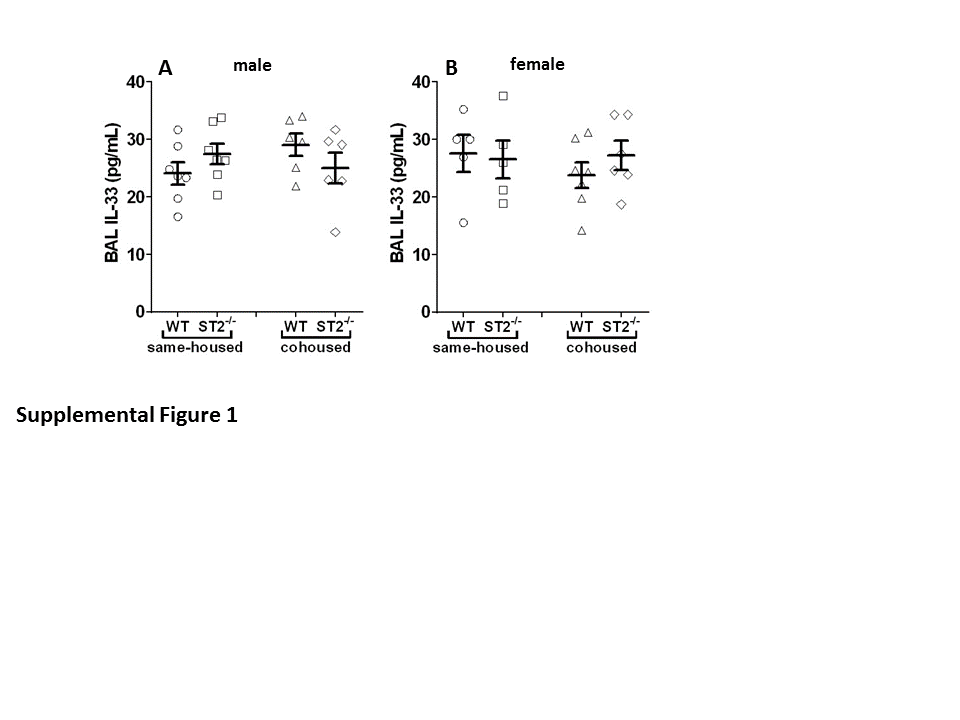


**Figure S1**: **BAL IL-33 in male and female mice.** BAL levels of IL-33 were measured in (A) male and in (B) female WT and ST2 deficient mice exposed to ozone. Mice were either housed with other mice of the same genotype (same-housed) or with mice with other genotype (cohoused). Each symbol denotes one individual mouse. Horizontal and vertical lines indicate mean and SEM respectively.
